# Genome Fusion Detection: a novel method to detect fusion genes from SNP-array data

**DOI:** 10.1093/bioinformatics/btt028

**Published:** 2013-01-17

**Authors:** Sebastian Thieme, Philip Groth

**Affiliations:** ^1^Department of Theoretical Biophysics, Humboldt-University of Berlin, 10115 Berlin, Germany and ^2^Therapeutic Research Group Oncology, Bayer Pharma AG, 13353 Berlin, Germany

## Abstract

**Motivation:** Fusion genes result from genomic rearrangements, such as deletions, amplifications and translocations. Such rearrangements can also frequently be observed in cancer and have been postulated as driving event in cancer development. to detect them, one needs to analyze the transition region of two segments with different copy number, the location where fusions are known to occur. Finding fusion genes is essential to understanding cancer development and may lead to new therapeutic approaches.

**Results:** Here we present a novel method, the Genomic Fusion Detection algorithm, to predict fusion genes on a genomic level based on SNP-array data. This algorithm detects genes at the transition region of segments with copy number variation. With the application of defined constraints, certain properties of the detected genes are evaluated to predict whether they may be fused. We evaluated our prediction by calculating the observed frequency of known fusions in both primary cancers and cell lines. We tested a set of cell lines positive for the BCR-ABL1 fusion and prostate cancers positive for the TMPRSS2-ERG fusion. We could detect the fusions in all positive cell lines, but not in the negative controls.

**Availability:** The algorithm is available from the supplement.

**Contact:** philip.groth@bayer.com

**Supplementary information:** Supplementary data are available at *Bioinformatics* online.

## 1 INTRODUCTION

### 1.1 Motivation

Genomic alterations are changes within the genomic sequence due to deletions, amplifications, translocations and other genomic rearrangements. These alterations can affect the balance of gene regulation networks and thus the proliferation and survival of cells (Deng *et al.*, 2011). In consequence, genomic alterations are often observed in cancer (Rajagopalan and Lengauer, 2004), now widely accepted as a disease with a strong genetic component based on the insight that cancer is almost always accompanied by rather severe changes on the genetic level (Mitelman *et al.*, 2007). Research in the field of oncogenomics focuses on such mutation events most likely to result in deregulation (i.e. inactivation or activation) of genes. Inactivation of tumor suppressor genes, for example, plays a major role in cancer development (Mitelman *et al.*, 2007).

The first detected functional fusion of two genes was found in chronic myeloid leukemia (CML). It is a genomic translocation, generated on chromosome 22 by a translocation of the long arm of chromosome 9 to chromosome 22, causing a truncated chromosome 22, the so-called Philadelphia chromosome, and an elongated chromosome 9. The resulting fusion is named after the two involved genes, BCR-ABL1. It was shown that this fusion plays an important role in the development of CML (Nowell and Hungerford, 1960). Even though the relevance of translocation events in cancer development was pointed out, it was widely assumed that this is a unique feature of leukemia (Edwards, 2010). One reason for this was the lack of technologies to detect complex structural rearrangements within the genome with high resolution. Hence, fusion genes remained unexplored for some time.

This view changed with the decoding of the human genome and the associated substantial progress in the development of laboratory and bioinformatics methods (Chen *et al.*, 2011; Kearney and Horsley, 2005). The advent of high-throughput methods like RNA-sequencing (RNA-seq) (Chen *et al.*, 2011) and high-resolution microarrays have enabled accurate descriptions of structural changes within the genome. This advancement in technology facilitated the detection of further fusions, e.g. the EML4-ALK fusion (Soda *et al.*, 2007) in lung cancer and the TMPRSS2-ERG fusion (Tomlins *et al.*, 2007) in prostate cancer. Their identification and characterization led to the conclusion that fusion genes also play an important role in solid cancers, dramatically changing the interest in fusion genes (Cazzaniga *et al.*, 2001; Edwards, 2010; Graux *et al.*, 2004). As a consequence, the available information on cancer genomes and their chromosomal aberrations has increased significantly in the past few years, leading to drugs for inhibiting and diagnostic kits for detecting them.

Pfizer, for example, developed Crizotinib, a drug to inhibit the ALK part of the EML4-ALK fusion (Bang, 2011). Novartis AG developed Imatinib (trade-name: Glivec), targeting the protein encoded by BCR-ABL1 and inhibiting the activity of the tyrosine kinase ABL1 (Ohm *et al.*, 2012). Recently, it was shown that Sorafenib (trade-name: Nexavar, developed by Bayer AG) also inhibits BCR-ABL1 kinase activities, including the Imatinib-resistant E255K and T315I mutants of this fusion (Kurosu *et al.*, 2009). The fusion of the TMPRSS2 gene with E-twenty six (ETS) family genes like ETV1, ERG and ETV4 occurs in up to 70% of all prostate cancers and is therefore a specific biomarker for prostate cancer diagnostics (Perner *et al.*, 2007). Companies like China Medical Technologies (http://www.chinameditech.com) and KREATECH (http://www.kreatech.com) have developed kits to detect the fusion of TMPRSS2 with an ETS family member, increasing the sensitivity of diagnosing prostate cancer (Perner *et al.*, 2007). The importance of research in this field is shown by the high success rate of these drugs and diagnostic kits.

### 1.2 Structural rearrangements

Structural or quantitative changes within the genome are so-called genomic aberrations. Changes within a chromosome are called chromosomal aberration. They can, for example, be detected by examining copy number variations (CNVs). A CNV is a change in the copy number (CN) of genomic segments caused by evolutionary events like deletions, amplifications or translocations (Conrad *et al.*, 2010). Such kinds of aberrations frequently occur during cancer development and are often unbalanced, meaning that there is a quantitative change in genetic material caused by a mutation event (Johansson *et al.*, 1996). The transition region of segments with CNVs is defined as a change in the CN of two neighboring genomic segments within a chromosome. It is often referred to as a breakpoint (Ritz *et al.*, 2011). Notably, by this definition, a breakpoint is only found in unbalanced fusion events. It has been proposed that balanced events most likely occur as early changes, whereas later aberrations in substantial tumors are typically unbalanced (Johansson *et al.*, 1996).

Genomic aberrations can be more complex than deletions or amplifications. Consider as an example two genes at different breakpoints, originally coding for two different proteins, merging to a fusion. The resulting sequence contains the entire or partial information of both genes, which may lead to a protein with novel functions (Long, 2000). A fusion causes the change of the physical genomic position therefore loss of the regulatory elements of this gene. This may, for example, change the expression of the involved gene (Huang *et al.*, 2012). The fusion gene TMPRSS2-ERG, for example, results from a deletion of three million base pairs. Due to the fusion event, the ERG gene is regulated by the TMPRSS2 promoter, leading to overexpression of ERG (Tomlins *et al.*, 2005). However, most of the occurring fusion genes are non-functional (loss of function of one or both genes). The functionality of a fusion gene depends on certain cell properties like the cell type (tissue specificity), the cell stage (replication activity) and the mode of action of the translated protein. In addition, the fusion gene has to fulfill certain properties to encode a functional mRNA, e.g. a cap structure for transport into the cytoplasm, a poly-A tail for the translational process and the ability to fold into a 3D structure (Salim *et al.*, 2011; Yanai *et al.*, 2002).

### 1.3 Related works

Ritz *et al.* developed the algorithm ‘Neighborhood Breakpoint Conservation’ (NBC) (Ritz *et al.*, 2011), which uses the CNV information from array comparative genomic hybridization (aCGH) (Pinkel and Albertson, 2005) to calculate breakpoints, a required prerequisite for predicting fusion genes on DNA level. The output of NBC comprises common breakpoints or pairs of common breakpoints in a given sample calculated on the basis of Bayesian statistics. The Bayesian statistic uses conditional probability to give evidence about the plausibility of an event. The resulting pairs of common breakpoints can then be used for fusion detection. The algorithm calculates all possible CN profiles (genomic segmentations), from which breakpoints could be derived. Next, it calculates the probability of a breakpoint between two probes, considering all possible CN profiles of an individual. In the final step, NBC combines the calculated probabilities of all breakpoints from each individual to find recurrent breakpoints across all individuals. The results are split into sets of either single breakpoints or pairs of breakpoints of a probe or an interval. Ritz *et al.* use all identified CN profiles of an individual to detect breakpoints with a certain probability. Owing to this approach, the variability in the position of a breakpoint within a gene or loci can be considered. Hence, pairs of common breakpoints can be used to detect fusion genes with positional variability. The advantage of this method is the ability to find functional and silent fusions. The main disadvantage of using aCGH microarrays is their low resolution compared with other technologies, e.g. RNA-seq. Given that CNVs can be located in small regions of a few hundred base pairs, they will not be detectable by methods relying only on low resolution (Perry *et al.*, 2008).

### 1.4 Contribution

We present Genomic Fusion Detection (GFD), an algorithm to detect fusion genes on DNA level based on segmentation data from high-resolution Affymetrix Genome-Wide Human Single Nucleotide Polymporism (SNP) Array 6.0 (SNP6) data. As input, we use here segmentation data calculated with the previously published tool PICNIC (Greenman *et al.*, 2010), based on a Bayesian Hidden Markov Model (HMM). These data are then processed within GFD in three steps. In the first step, breakpoints are detected in the predicted segmentations. In the second step, several constraints are applied to detect fusion genes with certain properties. In the final step, all samples are scanned for common fusion predictions to reduce false-positive predictions.

## 2 MATERIALS AND METHODS

### 2.1 Cancer cell lines

We use Affymetrix Genome-Wide Human SNP6 data of 13 cell lines from three different tissues and different cancer subtypes and of 82 primary prostate samples. Cell line data were obtained from the Gene Expression Omnibus (GEO; http://www.ncbi.nlm.nih.gov/geo) (Accession: GSE36138) and the Wellcome Trust Sanger Institute (http://www.sanger.ac.uk). The datasets were divided into two subsets. The first subset (Supplementary Table S1) is a collection of seven BCR-ABL1–positive CML cell lines (BV-173, EM-2, K-562, LAMA-84, MEG-01, JK-1, KCL-22) and one BCR-ABL1–negative AML cell line (KG-1) (fusion state according to Mahon *et al.*, 2000; Palsson and Masters, 2010). The second subset (Supplementary Table S2) consists of 44 primary prostate cancer and 38 matched normal samples of two different ethnic backgrounds (39 Chinese; 5 UK), of which one Chinese and four UK samples are TMPRSS2-ERG–positive according to the source (Mao *et al.*, 2010) (GEO accession: GSE18333). As reference, six prostate cancer cell lines (22Rv1, DU145, LNCaP, NCI-H660, PC3, VCaP) were included (retrieved from the sources referenced above). Each sample is represented by the data of the respective SNP6 experiment. RNA-seq data for cell line K-562 was downloaded from the NCBI SRA Database (http://www.ncbi.nlm.nih.gov/sra) (Accession: SRR018269).

### 2.2 deFuse

deFuse was developed by McPherson *et al.* (McPherson *et al.*, 2011). This algorithm detects fusion genes in RNA-seq data. Beside clearly aligned paired-end reads, ambiguously aligned paired-end reads (see e.g. Fullwood *et al.*, 2009; Hall and Quinlan, 2012; Maher *et al.*, 2009 for details on these concepts) are also considered and the most likely alignment position of such reads is calculated. deFuse is able to identify gene fusions with boundaries between known exons as well as between intronic or intergenic sequences. For validating fusions, several confidence measures estimating the correctness of each prediction are implemented.

### 2.3 Copy number prediction

Copy Number Analyzer for GeneChips (CNAG) (Nannya *et al.*, 2005), dChip (Zhao *et al.*, 2004) and other tools (Korn *et al.*, 2008; Wang *et al.*, 2007) predict CN (see the review by Pinto *et al.*, 2011). Most of them use normal tissue as baseline. However, CNV in cancer samples is typically increased (Cappuzzo *et al.*, 2005; Suzuki *et al.*, 1997). Given this increase, most available tools are not suited to detect CNVs in cancer samples (Lamy *et al.*, 2011; Li *et al.*, 2010). In contrast, PICNIC (http://www.sanger.ac.uk/genetics/CGP/Software/PICNIC/) considers the increased CN in cancer and is therefore our method of choice (Greenman *et al.*, 2010).

### 2.4 Genomic Fusion Detection

#### 2.4.1 Summary

**Fig. 1.**
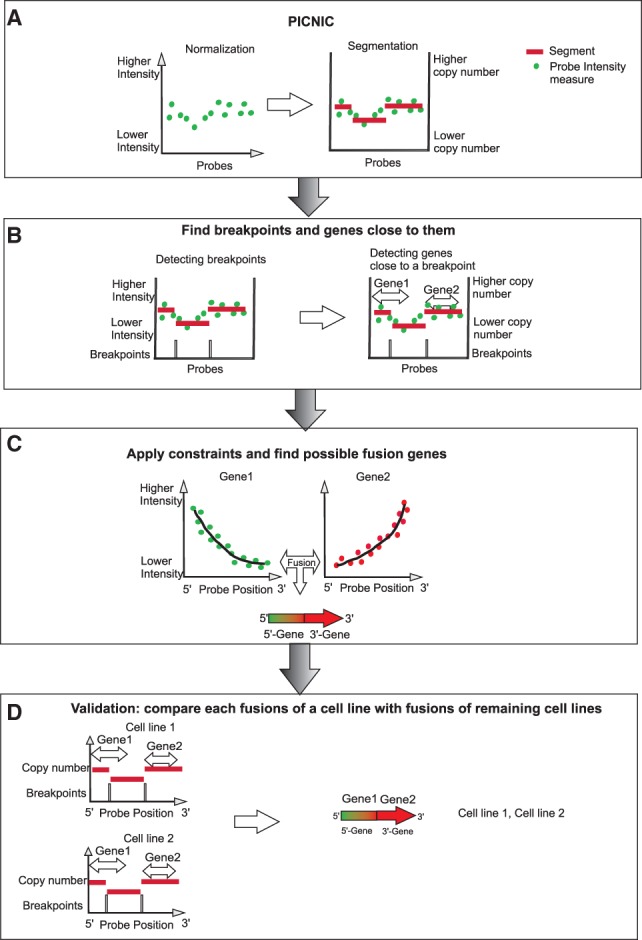
(**A**) Pre-processing step: PICNIC normalizes and segments the data. (**B**) Determine breakpoints within the predicted segmentation, delete artifacts and find genes close to a breakpoint. (**C**) Find gene combinations, fulfilling the constraints. (**D**) Compare the results of each sample with results of all processed samples to find common fusion events

Our GFD algorithm consists of one pre-processing and three main steps (Fig. 1). In the pre-processing step, SNP6 data are processed by PICNIC (Release Nov 2010) to get the segmentation of the data. Our algorithm takes this information from PICNIC as input (Fig. 1A). It is used to predict fusion genes. The input data are processed in three steps. In the first step, breakpoints are determined in the segmentations, artifacts are deleted and genes located close to a breakpoint are identified (Fig. 1B). Then, gene pairs fulfilling the required constraints are detected (Fig. 1C). In the last step, the result for each sample is compared with the results of all processed samples to find common fusion events and reduce false-positive predictions (Fig. 1D). GFD is based on the ideas of Ritz *et al.*, extending them by the following features (see Supplementary Table S3 for details).

#### 2.4.2 Filtering and selecting gene candidates

In this step, the segments predicted by PICNIC are selected and the breakpoints on each chromosome are detected. Segments represented by a single probe are hard to discern from experimental or algorithmic artifacts. Hence, they are removed to minimize prediction errors. An artifact is, for example, characterized by adjacent probes belonging to larger segments with a differing CN than their surroundings, but similar CN to each other. For example, the artifact could have a CN of 5, but the respective segment a CN of 2.

#### 2.4.3 Selecting genes closest to a breakpoint

We define that a fusion gene consists of a 5′-part of the fusion (upstream gene, or 5′-gene) and a 3′-part of the fusion (downstream gene, or 3′-gene). The Genome Reference Consortium Human Reference 37 (GRCh37, Ensembl rev 65 of Dec 2011) was used for predicting fusions. Each involved gene must be adjacent to a breakpoint. On the forward strand, the 3′-part of a fusion occurs downstream of a breakpoint and the 5′-part of a fusion occurs upstream and vice versa on the backward strand.

Intervals surrounding a breakpoint are scanned for genes out of a set of 21 991 unique Ensembl genes. Two cases are considered how a breakpoint can be observed in respect to a gene. First, a gene can stretch across one or more breakpoints, dividing the gene into subsegments. In this case, each of the subsegments may yield a different CN. If two subsegments are observed, each will be assigned to a 5′-gene or a 3′-gene. If there are three or more subsegments, the most upstream subsegment will be classified as a 5′-gene and the most downstream subsegment as a 3′-gene, whereas inner subsegments are assigned to both groups. Alternatively, the sequences of two genes can be fused entirely. Here, it is defined that the distance of each gene to the nearest breakpoint must be at most 10 kb. Typically, the average distance of human introns is 5 kb with a standard deviation of 4.7–24 kb (Sakharkar *et al.*, 2004). Therefore, a non-coding sequence of at most 20 kb between both genes describing a fusion is feasible. This ensures that the distance between both genes is statistically not too long to enable function. Based on this constraint, all fusions not nearby the two sides of breakpoints will be missed by GFD. This is based on the biological assumption that ‘breakpoints point to the location of directly cancer-relevant genes’ (Mitelman, 2005) and the technological limitation that only unbalanced fusions are detectable by SNP6 (Greisman *et al.*, 2011). Genes fulfilling one of these criteria are considered for the next steps. Once the most likely genes to enter a fusion have been selected, they are analyzed for the most likely pairing for a fusion, as not any two genes can form a functional fusion gene, e.g. two genes with opposing orientation. Therefore, further required constrains are described next.

#### 2.4.4 General required constraints

Several features are considered to identify the most likely gene pairs involved in a fusion. These features are the maximal difference of the total CN, gene orientation, fusion side, minimal and maximal length of a fusion and the tendency of probe intensities in both genes.

The probe intensity values used to calculate a specific CN show high variability. This fact is considered by the HMM in PICNIC. The transition between two CN states depends on the probability given in the HMM, resulting in a sequence of CN states based on the probe intensity values and the probabilities calculated for the transition. The CN sequence calculated by the Viterbi algorithm applied in the HMM returns the most likely CN sequence, but there is no guarantee that the calculated sequence is the true CN sequence. Thus, CNV depends on the probabilities given by the HMM and is therefore not exact. We take this variability into account. Still, because both genes of a fusion are physically joined, their CN should only deviate by a small value. We allow a CN difference of at most _±1_ here.

The gene end lying closest to the next breakpoint defines the fusion side. Thus, the orientation of the strand at this position is another constraint.

A further constraint is that the fusion gene must keep a certain length. Therefore, both a minimal and a maximal acceptable length for a fusion, described by the shortest and longest gene found in the gene collection are set as minimal and maximal accepted length of a fusion, respectively.

**Fig. 2.**
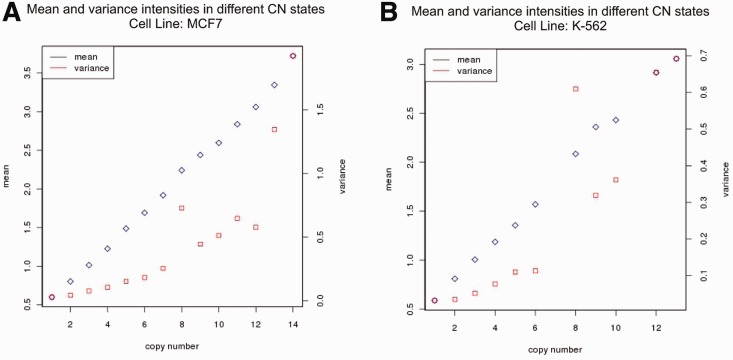
The variance of intensities within a CN state increases with the CN. Exemplarily shown for (**A**) cell line MCF-7 and (**B**) cell line K-562

If two genes are fused, they will be replicated together and we assume them to be amplified together. This leads to a constraint, i.e. the trend of intensities at the fusion region between the 5′-gene and the 3′-gene (border of a fusion or crossover region). This region is described by seven probes of each involved fusion subsegment (if a fusion subsegment has less than seven probes, it is ignored). Intensity values of these probes in the crossover region have to be similar. A closer look at any gene interval reveals that the variance of probe intensities increases proportionally to the CN, i.e. the variance of the intensities can vary in-between CNs and samples (Fig. 2). Hence, a simple comparison of average intensities is not possible. Instead, we calculate smoothed intensities with loess normalization, producing a smooth set of values from the intensity values with high variance. A window of pre-defined size is shifted across these and a polynomial function is fitted at each data point within the window (Mount, 2004).

Thus, our approach considers the difference in each gene interval’s variance. To optimize the smoothing, the span is calculated individually for each fusion candidate. The analysis performed here relies on the small-sample-size corrected Akaike information criterion (AICC) because it is robust and accounts for a bias in small datasets. The optimal span is the minimal span that best fits the polynomial function of the loess normalization. This optimization is done iteratively over an interval for 2%, 3%, and so forth, to 95% of the data points. The intensity trend in their hypothesized border region for both candidate genes for a fusion is the best indication for the feasibility of a fusion and can be described by Pearson correlation. Given that the border interval is supported by seven probes of each gene, Pearson correlation is comparable between possible gene combinations. If the absolute correlation between both intervals is >0.9, the gene pair is still a valid candidate. A gene pair fulfilling all above constraints is reported as fusion candidate and will be validated next.

#### 2.4.5 Evaluation of likely fusion candidates

**Fig. 3.**
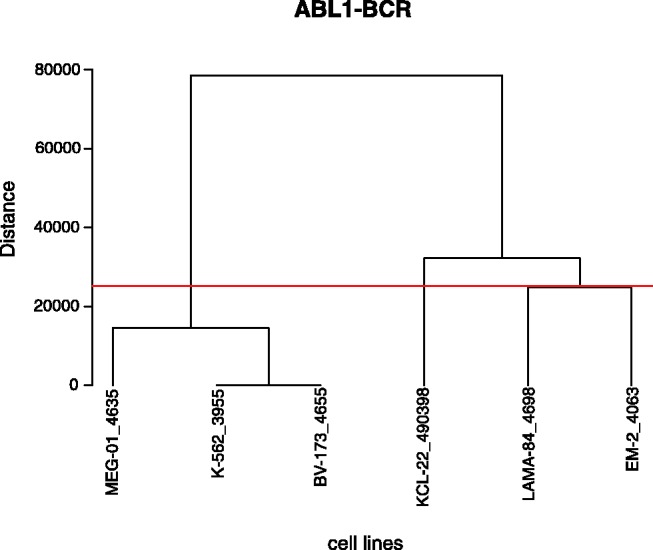
The dendrogram shows a visualization of the ABL1-BCR fusion’s hierarchical clustering. The horizontal line describes the threshold of 40% of the median sequence length

The previous analysis steps lead to a list of all possible fusion candidates for each sample. This list is scanned for candidates common to at least two samples, termed ‘group’ hereafter. Every group will be evaluated separately. To ensure the prediction of fusion genes with similar segment positions and sizes, the subsegments of a fusion of each group are filtered for similar start and end positions using the genomic positions of each fusion. Owing to variability of the segment positions within a fusion gene caused by breakpoints (truncated gene) within intron regions, at least two positions have to be equal, e.g. the start position of the 5′-gene and the end position of the 3′-gene over all group members. Next, the variability of all other positions is analyzed. Therefore, the Euclidean distance of the non-conserved positions of a fusion subsegment is calculated for the respective position across all group members. Hierarchical clustering with complete linkage is applied on the distance matrix to either classify the fusion into different subgroups, which are determined by the distance among each other, or to discard a sample because it does not contain the fusion. Additionally, the median sequence length of the group is calculated. Each fusion event will receive an individual range from calculating the median sequence length of this particular fusion event across all samples. Fusions with a distance >40% of the median sequence length to the other cluster members will be discarded. The threshold of 40% was chosen to ensure certain variability in fusion length, considering variability within the length of fusion genes resulting from breakpoints within intron regions. Only subgroups of at least two samples will be considered for further analysis (Fig. 3 for an example). The resulting groups and subgroups are predicted as fusion genes.

The root mean squared difference (RMS) of the smoothed intensities surrounding the fusion border is calculated as an additional feature. It describes the distance between the smoothed border intensities from the upstream and downstream gene partner of a fusion. If this value is close to zero, a consistent crossover between both genes is observed. If a higher RMS is observed, it may imply a false-positive fusion. However, the CNs of both genes have to be considered for interpreting the RMS because the intensity variance increases with increasing CN. Hence, if high CN is observed, a higher RMS can also imply a true-positive fusion yielding an additional parameter for evaluating fusion genes.

## 3 Results

The BCR-ABL1 fusion was predicted to exist in all BCR-ABL1–positive cell lines (Table 1), but not in the BCR-ABL1–negative cell line KG-1 (Supplementary Table S4), as there were no breakpoints in BCR or ABL1 detected (Supplementary Table S5). Both segments of the BCR-ABL1 fusion, within the BCR-ABL1–positive cell lines differ in their CN. The CN of both segments is inconsistent across the cell lines. Most of them have a CN between two and five except K-562, which sets itself apart from the others with a CN of 13 for the BCR part and a CN of 12 for the ABL1 part. The absolute correlation coefficient is 0.999 for the BCR-ABL1 fusion in all cell lines. An RMS of 0.7 can be observed for the crossover region of the BCR-ABL1 fusion in K-562 owing to the high CN. In the other cell lines, the RMS is smaller. Given that the BCR-ABL1 fusion results from a translocation of the long arm of chromosome 9 to chromosome 22 and vice versa, there is a BCR-ABL1 fusion on chromosome 22 and an ABL1-BCR fusion on chromosome 9. The ABL1-BCR fusion was predicted in five of seven BCR-ABL1–positive cell lines. The CN of all predictions is between one and three with a correlation coefficient of at least 0.98. It can be observed that the CN of both parts from the ABL1-BCR fusion is continuously lower than in the CML-characteristic fusion BCR-ABL1. This is especially true for the CN of the ABL1-BCR fusion in K-562, which has a CN of three for the ABL1 and two for the BCR part and a RMS of 0.7.

**Table 1.** BCR-ABL1 fusion

Cell line	RMS	Correlation	CN BCR	CN ABL1
BV-173	0.4547	0.9994	3	2
EM-2	0.1439	−0.9995	5	4
JK-1	0.1974	0.9994	3	3
K-562	0.7057	0.9999	13	12
LAMA-84	0.3966	−0.9999	5	4
MEG-01	0.2731	−0.9996	4	4
KCL-22	0.2846	−0.9998	3	3

Prediction of the BCR-ABL1 fusion in cell lines. The 5′-gene and 3′-gene of the fusion corresponds to the BCR and ABL1 segment, respectively.

The ABL1-BCR fusion was predicted in six cell lines shown in Figure 3, but there are only two subgroups with three and two members reported (Table 2) because the cell line KCL-22 is discarded owing to the constraint that the distance between two fusions of one group has to be at most 40% of the median sequence length.

**Table 2.** ABL1-BCR groups

Cell line	Start position ABL1	End position ABL1	Start position BCR	End position BCR	Group
BV-173	133 592 718	133 605 045	23 633 005	23 650 421	1
K-562	133 592 718	133 605 045	23 633 005	23 650 421	1
MEG-01	133 592 718	133 619 550	23 632 191	23 650 421	1
EM-2	133 592 718	133 658 924	23 635 685	23 650 421	2
LAMA-84	133 592 718	133 683 574	23 633 005	23 650 421	2

Prediction of the ABL1-BCR fusion subgroups. It should be noted that start and end positions are based on ENSEMBL version 65 and may change slightly in other genome references without an effect on the prediction.

Table 2 also shows that the start and end positions of this fusion are conserved, whereas the positions within the fusion border vary. The positions of BV-173, MEG-01 and K-562 lie close together, whereas the positions of EM-2 and LAMA-84 differ from the other cell lines. This observation confirms the clustering analysis in Figure 3. The main differences are in the end position of the ABL1 part, leading to two groups of ABL1-BCR fusions in the clustering.

To estimate the quality of SNP6-based fusion prediction, the results of GFD are compared with deFuse, an algorithm detecting fusion proteins in RNA-seq data. deFuse detected 11 fusions in cell line K-562, but only the BCR-ABL1 fusion was detected by both algorithms. The start and end position of the BCR segment differ ∼4 kb and ∼87 bp, respectively. The ABL1 segment differs ∼119 kb at the start position and ∼1.5 kb at the end position. The fusion length is 258 798 bp for the GFD prediction and 144 320 bp for the deFuse prediction, which is a difference of 114 478 bp. deFuse did not find the ABL1-BCR fusion. Probe intensities of the BCR and the ABL1 gene in K-562 are shown in Supplementary Figure S1. The main parts of BCR (80 probes) and ABL1 (245 probes) define the BCR-ABL1 fusion, whereas the ABL1-BCR fusion is defined by the smaller remaining parts [17 probes (ABL1) and 20 probes (BCR)]. Three other interchromosomal fusions were found in two BCR-ABL1–positive cell lines, but none in all (Supplementary Table S4). No common intrachromosomal fusion was found. BCR-ABL1–negative KG-1 has no fusion in common with any other cell line (Supplementary Table S4).

**Fig. 4.**
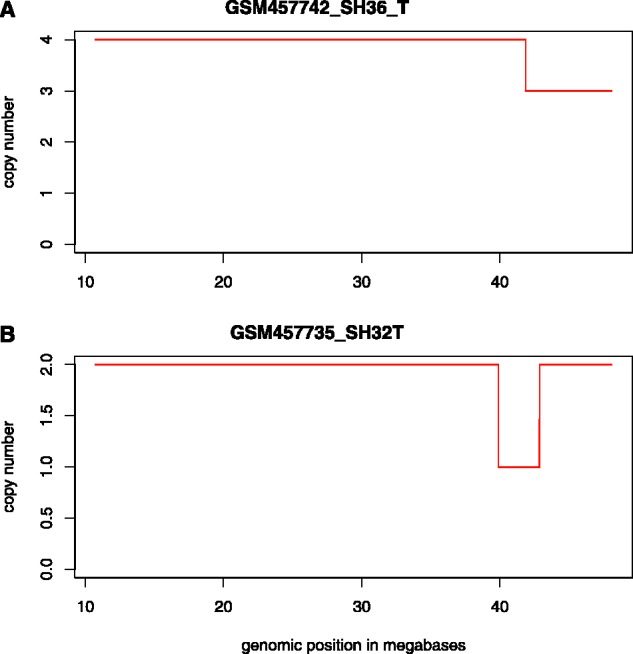
Stretch across chromosome 21 showing deletions in two primary prostate cancer samples. SH32 yields the characteristic deletion required to form the TMPRSS2-ERG fusion, whereas SH36 has only a partial deletion

We found the TMPRSS2-ERG fusion in NCI-H660, but not in any other prostate cancer cell line (Supplementary Table S6). In the primary prostate samples, we verified the fusion in all fusion-positive samples (i.e. SH32, P9, P55, P68 and P98), but not in any of the matched normal samples or in sample SH36 (Supplementary Table S6). The latter harbors a deletion in 21q22.3, but not across 21q22.2 and 22.3 required to constitute the fusion (Fig. 4).

**Fig. 5.**
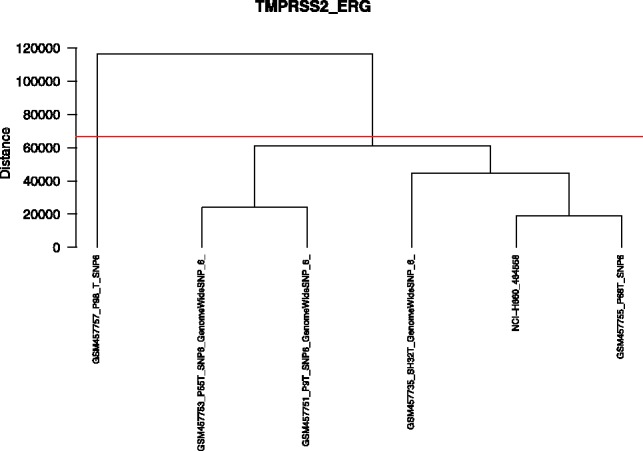
The dendrogram shows the TMPRSS2-ERG fusion’s hierarchical clustering in primary prostate cancer samples and a cell line. The horizontal line describes the threshold of 40% of the median sequence length. Sample P98 deviates from this threshold and is therefore considered an outlier

One of the primary prostate cancer samples is seen as outlier in the clustering (Fig. 5) due to a violation of a constraint (>40% of the median sequence length and no other sample with a fusion of similar length). Here, we found an atypical stretch of the fusion across both genes not seen in any other sample.

## 4 Discussion and Conclusions

Here we present for the first time our novel development, the GFD algorithm. This algorithm uses the high resolution of SNP6 microarrays for detecting gene fusions. The GFD algorithm detects functional fusion genes from unbalanced mutation events in cancer. We show that the algorithm’s accuracy is comparable with deFuse, detecting fusion genes from RNA-seq data. The comparison showed that the BCR-ABL1 fusion can be found with both approaches. Differing start positions of ABL1 can be explained by the missing intron sequences within the data used by deFuse. From this, it follows that both approaches predict the same BCR-ABL1 fusion. The ABL1-BCR fusion was only predicted by GFD because the ABL1-BCR fusion is typically not expressed (Uphoff *et al.*, 1999). Owing to the fundamental differences in data types, it is not our scope to benchmark DNA-microarrays against RNA-seq. Still, using a high-resolution DNA-microarray enables predicting fusion genes that are not expressed, i.e. not detectable by RNA-seq. Deep next-generation sequencing may alter this situation, but the cost-advantage of our approach is currently >10-fold in contrast. In addition, GFD is able to detect non-functional, silenced and novel fusions.

For evaluation, seven BCR-ABL1–positive CML cell lines (Mahon *et al.*, 2000; Uphoff *et al.*, 1999) and one BCR-ABL1–negative AML cell line (Hochhaus *et al.*, 1996) were used. It was shown that GFD predicts the BCR-ABL1 fusion in all BCR-ABL1–positive cell lines. The breakpoint positions of BCR-ABL1 within cell line K-562 on chromosome 22 and chromosome 9 found by GFD are close to the published positions (Shibata *et al.*, 2010). The ABL1-BCR fusion formed on chromosome 9 was predicted in five of seven cell lines. There is a chance that a gene will still be normally expressed even though it participates in a fusion, owing to a second copy of the same gene that is not involved in a fusion (Uphoff *et al.*, 1999). This could explain the CN difference within the segments of the BCR-ABL1 and ABL1-BCR fusions. In the EM-2 cell line, the CN of the BCR part in both fusions is higher than CN of the ABL1 part in both fusions. This can also be observed within the BCR-ABL1 and ABL1-BCR fusion in K-562. However, in this case, an RMS of 0.7 is observed in both fusions. The difference in the border region indicates least one BCR and one ABL1 gene expressing normal transcripts.

As expected, TMPRSS2-ERG fusions could not be detected in negative prostate cancer cell lines PC3, LNCaP, DU145 and 22Rv1, but rather unexpectedly also not in VCaP. This cell line is known to harbor at least one normal TMPRSS2 and ERG gene, making it difficult to study the fusion *in vitro* (Mertz *et al.*, 2007). In a clinical dataset of 82 primary prostate samples, we could successfully identify all previously described fusion-positive samples. One of these samples contains the typical deletion, but also unusually long parts of both fusion partners, rendering it an outlier.

We have shown that GFD is able to detect functional fusion genes, deriving from unbalanced mutation events in cancer. In addition, we have shown that GFD has a similar accuracy as an approach to detect fusion genes based on RNA-seq. A future application of GFD could be combined run with an RNA-seq-based algorithm. In this case, one can distinguish between functional and non-functional fusions. Also, transcriptional and translational position information of the fusion could be gained. Thus, it would become possible to predict post-transcriptional and balanced fusions as well as unbalanced, silenced and non-functional fusions.

*Funding:* We acknowledge funding of the German Federal Ministry of Education and Research German Federal Ministry for Education and Research (Bundesministerium für Bildung und Forschung - BMBF): MedSys projects 0315428F and 0315416B.

*Conflict of Interest:* none declared 

## Supplementary Material

Supplementary Data
