# Supplementary material for: Genome Fusion Detection: a novel method to detect fusion genes from SNP-array data
Source: Bioinformatics. 2013 Jan 17;29(6):671–7. doi: 10.1093/bioinformatics/btt028 (PMC3597144; doi:10.1093/bioinformatics/btt028)
Supplement: Supplementary Data [file supp_29_6_671__index.html]

Genome Fusion Detection: A novel method to detect fusion genes from SNP-array data — Genome Fusion Detection: a novel method to detect fusion genes from SNP-array data — Genome Fusion Detection: a novel method to detect fusion genes from SNP-array data — Supplementary Data 

# Genome Fusion Detection: a novel method to detect fusion genes from SNP-array data

## Supplementary Data

files

**Files in this Data Supplement:**

- Supplementary Data - zip file
- Supplementary Data - zip file
- Supplementary Data - zip file
