# Supplementary figures and images for: Genome Fusion Detection: a novel method to detect fusion genes from SNP-array data
Source: Bioinformatics. 2013 Jan 17;29(6):671–7. doi: 10.1093/bioinformatics/btt028 (PMC3597144; doi:10.1093/bioinformatics/btt028)

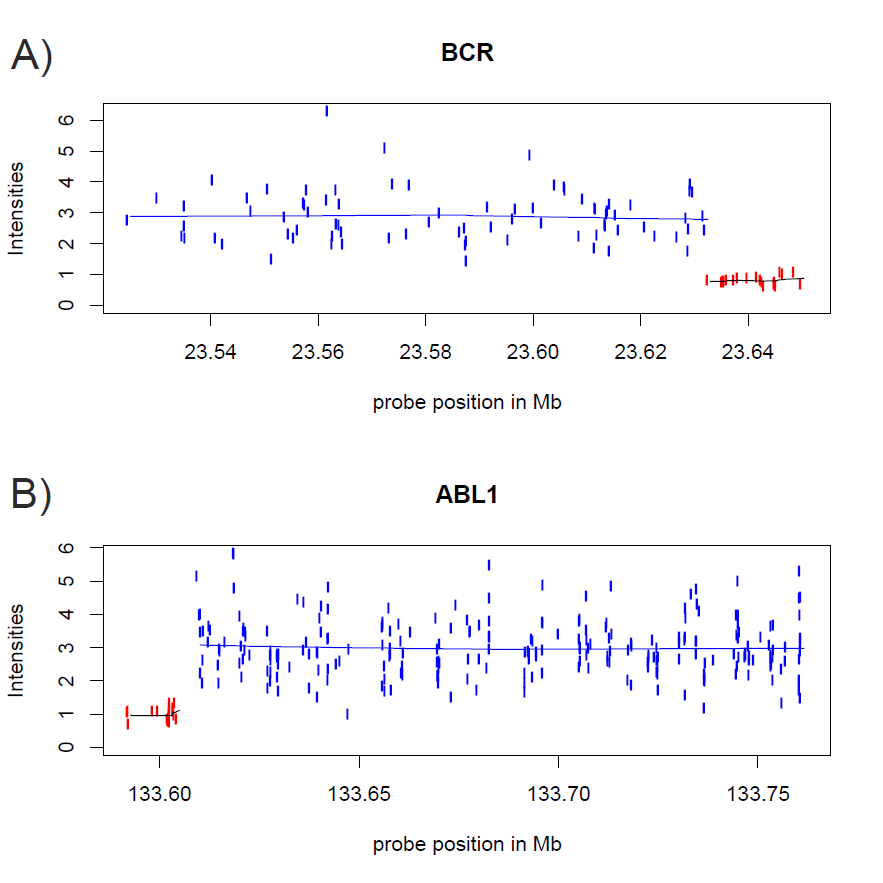

Supplement: Supplementary Data [file supp_btt028_supplementary_figures.zip › Supplementary_Figure_1.png]
